# Conventional analysis methods underestimate the plant-available pools of calcium, magnesium and potassium in forest soils

**DOI:** 10.1038/s41598-020-72741-w

**Published:** 2020-09-24

**Authors:** Bel Jérémie, Legout Arnaud, Saint-André Laurent, J. Hall Steven, Löfgren Stefan, Laclau Jean-Paul, van der Heijden Gregory

**Affiliations:** 1 INRAE Grand-EST Nancy, UR 1138 Biogéochimie des Ecosystèmes Forestiers, Route d’Amance, 54280 Champenoux, France; 2 Department of Ecology, Evolution, and Organismal Biology, Iowa State University, 251 Bessey Hall, Ames, IA 50011 USA; 3 Department of Aquatic Sciences and Assessment, Swedish University of Agricultural Sciences, P.O. Box 7050, 750 07 Uppsala, Sweden; 4 CIRAD, UMR 210 ecologie fonctionnelle et biogéochimie des sols et des agro-écosystèmes, Campus SupAgro, Bâtiment 12 - 2 place Viala, 34060 Montpellier Cedex 2, France

**Keywords:** Biogeochemistry, Environmental sciences

## Abstract

The plant-available pools of calcium, magnesium and potassium are assumed to be stored in the soil as exchangeable cations adsorbed on the cation exchange complex. In numerous forest ecosystems, despite very low plant-available pools, elevated forest productivities are sustained. We hypothesize that trees access nutrient sources in the soil that are currently unaccounted by conventional soil analysis methods. We carried out an isotopic dilution assay to quantify the plant-available pools of calcium, magnesium and potassium and trace the soil phases that support these pools in 143 individual soil samples covering 3 climatic zones and 5 different soil types. For 81%, 87% and 90% of the soil samples (respectively for Ca, Mg and K), the plant-available pools measured by isotopic dilution were greater than the conventional exchangeable pool. This additional pool is most likely supported by secondary non-crystalline mineral phases in interaction with soil organic matter and represents in many cases (respectively 43%, 27% and 47% of the soil samples) a substantial amount of plant-available nutrient cations (50% greater than the conventional exchangeable pools) that is likely to play an essential role in the biogeochemical functioning of forest ecosystems, in particular when the resources of Ca, Mg and K are low.

## Introduction

Magnesium (Mg), calcium (Ca) and potassium (K) are three major and essential nutrients for plants^1^. In forest ecosystems, their plant-available pools are assumed to be stored in the soil as dissolved cations in solution and as exchangeable cations adsorbed on the cation exchange complex^2,3^. This exchangeable pool is commonly measured in soil samples from an extraction with a concentrated salt reactant (e.g. NH_4_Cl, BaCl_2_, etc.). Because, in most cases, fertilization and liming are not common practices in forestry, plant-available nutrient cations are, on the long term, mainly supplied by atmospheric deposition and mineral weathering and vary as a function of the net nutrient losses from the ecosystems: mainly harvested biomass exportation and nutrient leaching below the rooting zone.

Forest ecosystems are often developed on poorly fertile soils where the plant-available pools of nutrient cations are frequently very low^4^. In the context of global change, the sustainability of forest ecosystems and their chemical fertility is highly at risk due to increasing nutritional, sivilcultural and/or climatic pressures. Despite the decreasing acidity of atmospheric inputs since the 1980s in Europe and North America, many forest soils are still suffering from on-going acidification^5–8^. In many cases, atmospheric inputs of nutrient cations have decreased over the past decades^7,9–12^. Finally, nutrient outputs from the ecosystem are often increasing due to the intensification of silvicultural practices (biomass export, etc.) to meet the increasing demand for wood biomass. These practices may severely impact soil fertility^13^, especially in forest ecosystems where the nutrient level is low^14^. As a result, over the last decade degradation of forest soil fertility have been reported worldwide and are expected to increase^6,15–17^.

It is however not yet fully understood how trees cope with very low nutrient resources in the soil and sustain long term forest productivity^18^. Numerous studies have reported significant discrepancies between modelled and empirically measured changes in plant-available pools of nutrient cations in the soil: mass balance and dynamic models tend to exaggerate nutrient cation depletion from the soil^19^ compared to measured changes in soil available pool between two dates^20^. In addition, many studies have reported discrepancies between chemical fertility (exchangeable pools, nutrient fluxes), tree nutrition (foliar nutrient concentrations) and forest productivity indicators^21–25^.

To explain these discrepancies, it has been hypothesized that the plant-available pool of nutrient cations may be larger than the exchangeable pool^20,26–28^. Aluminium and iron (hydr)oxides as well as amorphous aluminosilicate structures, which are abundant in acidic soils, may develop a cationic exchange capacity and adsorb cations^29^. The amorphous nature and the dynamic dissolution/precipitation of these structures may cause the adsorbed cations to become temporally occluded^30,31^. In a highly weathered tropical soil, Hall and Huang^32^ showed that a significant amount of Ca, Mg, and K was sequestered in iron (hydr)oxide secondary mineral phases. Moreover, potassium stored in part as “non-exchangeable” or fixed potassium^33^ (held between adjacent tetrahedral phyllosilicate layers of micas and 2:1 clay minerals such as vermiculite or illite) may be available to plant uptake. The intensity of “non-exchangeable” K release has been related to root absorption and root activity^34^ and may be a quite significant source of K for plant nutrition^35^.

Quantifying plant-available pools in the soil through soil extraction methods is challenging because it is difficult to define a chemical reagent with the same nutrient extraction potential as plant roots and their associated microorganisms. Nevertheless, the isotopically exchangeable pool (noted E_K_, E_Ca_ and E_Mg_) quantified by the isotopic dilution method has been shown to be the most adequate approach to quantify the plant-available pools^36,37^. Using radio-isotopes, previous studies found that the K and Ca isotopically exchangeable pool was larger than the exchangeable pool (over 45 agricultural soil studied)^38–43^. Two recent studies^44,45^, focused on soil samples from the Breuil-Chenue experimental forest and using a stable isotopic dilution approach, (i) showed that soil pools of Mg, Ca and K in addition to the exchangeable pool significantly contributed to chemical equilibrium reactions between the liquid and solid phases of the soil, and (ii) suggested that these pools were supported by secondary non-crystalline mineral phases such as Al and Fe (hydr)oxides, and amorphous aluminosilicates.

The objectives of this study are twofold: (i) to test the hypothesis that nutrient cation pools other than the conventional exchangeable pool contribute to solid solution equilibrium in a variety of forest soils covering a wide range of climatic, edaphic, chemical fertility and tree species cover conditions; (ii) to characterize the variability of the isotopically exchangeable pools in relation to the physical and chemical properties of the soil in order to better describe the different soil phases that may act as a support for this additional plant-available pool.

## Results

### Isotopically exchangeable pools

**Figure 1:**
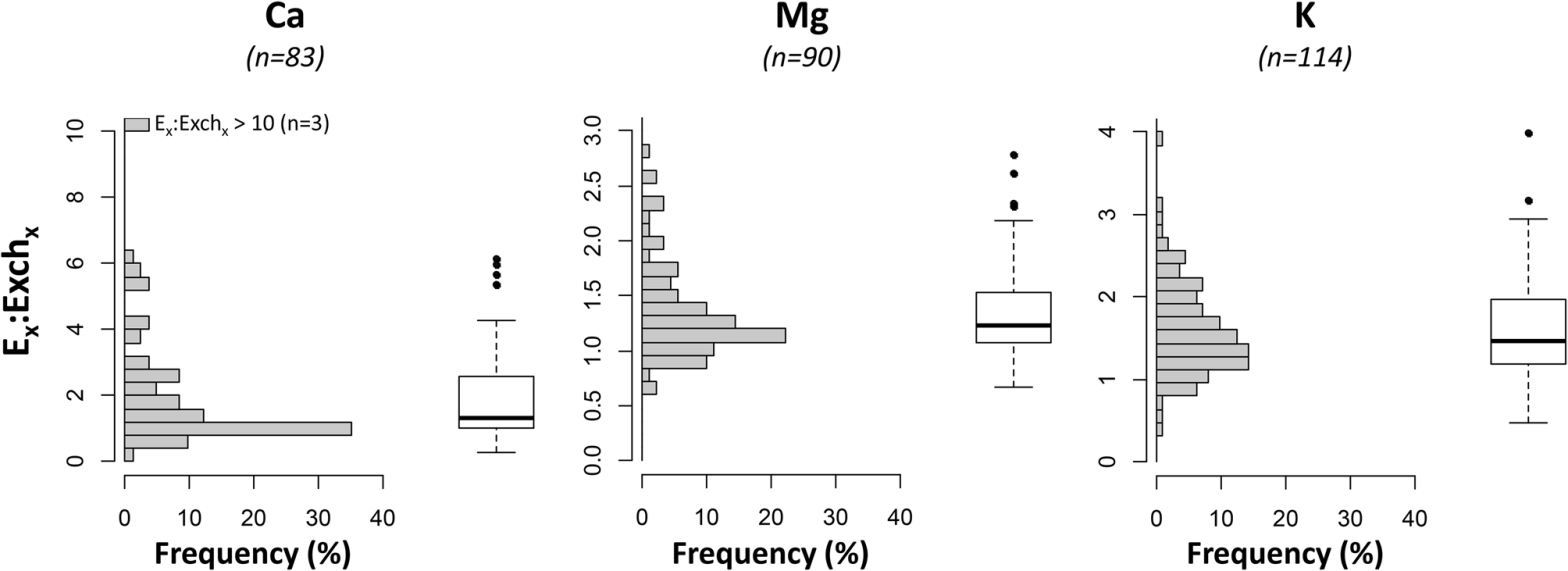
Histogram and boxplot of the distribution of the E_x_:Exch_x_ ratios for Ca, Mg and K. E_x_ represents the isotopically exchangeable pool and Exch_x_ the conventional exchangeable pool. The number of validated samples (n) is given in brackets.

**Figure 2:**
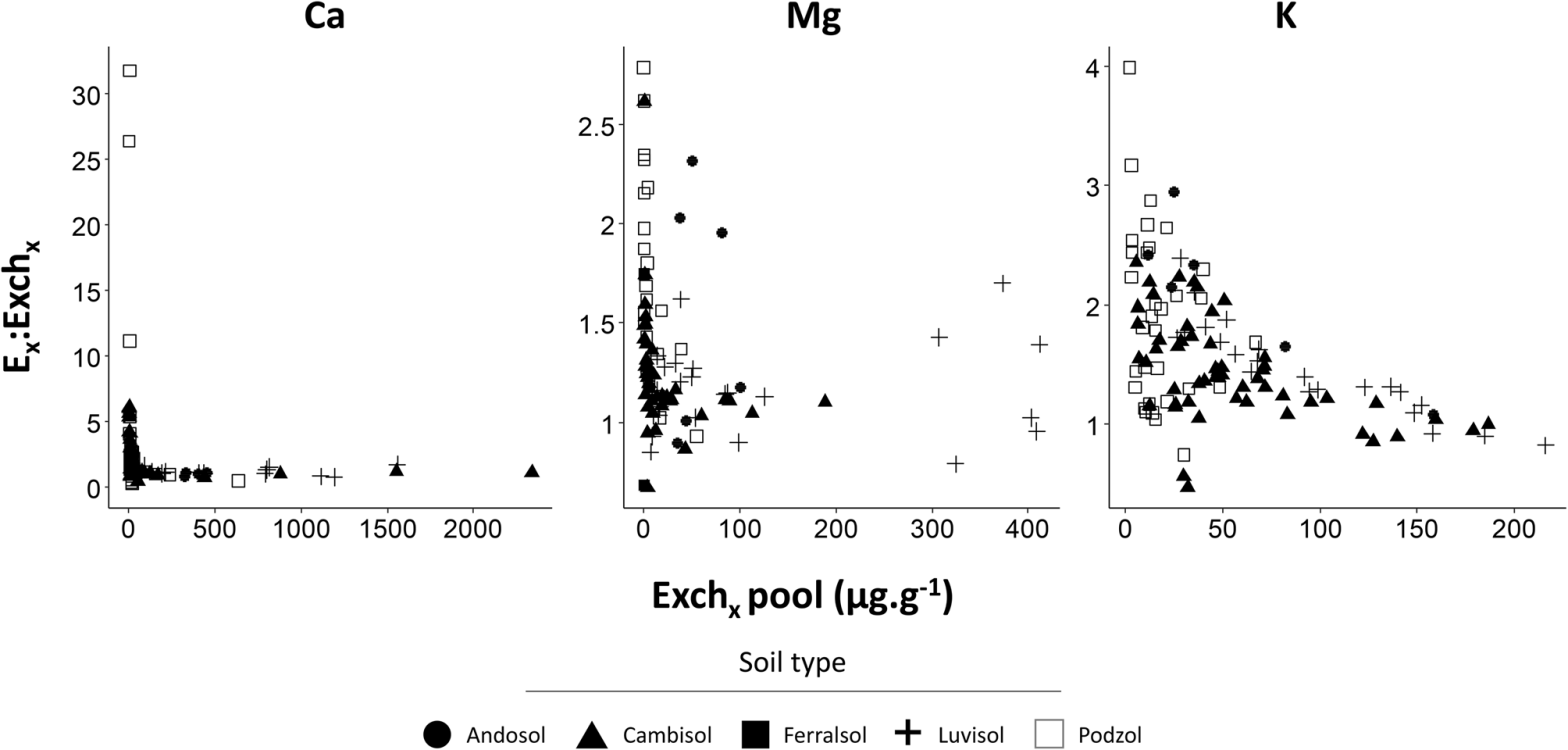
Relationship between the E_x_:Exch_x_ ratio and the conventional exchangeable (Exch_x_) pools for Ca, Mg and K.

The ratio of the isotopically exchangeable over the conventional exchangeable pool (E_x_:Exch_x_) ranged from 0.28 to 31.76 for Ca, from 0.67 to 2.79 for Mg and from 0.47 to 3.99 for K (Fig. 1, for individual results Table S3 supplementary data) but E_Ca_, E_Mg_ and E_K_ were larger than their respective exchangeable pool for the great majority of the soil samples: 81%, 87% and 90%, respectively, of soil samples had E_x_:Exch_x_ ratios above 1. The median ratios were respectively 1.30, 1.23 and 1.47 for Ca, Mg and K. The E_Ca_:Exch_Ca_ ratio was greater than 2 for one third of the samples and greater than 3 for 17%. For Mg and K, the E_X_:Exch_X_ ratio was greater than 2 for fewer samples compared to Ca: 10% and 24% respectively. The E_X_:Exch_X_ was higher for the samples with low Exch_X_ pools (Fig. 2). Over the entire dataset, the difference between the E_X_ and Exch_X_ pools was statistically significant (paired Wilcoxon-Mann–Whitney test) for all three elements (p-value < 0.01 for K and Mg and p-value < 0.05 for Ca), although homoscedasticity was not verified for Mg.

The repeatability of E_X_ measurements, estimated as the relative standard deviation was 16%, 14% and 8% for Ca, Mg and K respectively. Even when taking this analytical uncertainty into account, 58% (Ca), 62% (Mg) and 88% (K) of the samples had an E_x_-pool significantly greater than the Exch_x_ pool (data not shown). A small proportion of samples had E_x_-pools lower than the Exch_x_ pools (E_x_:Exch_x_ < 1): 19%, 13% and 10% for Ca, Mg and K respectively.

**Figure 3:**
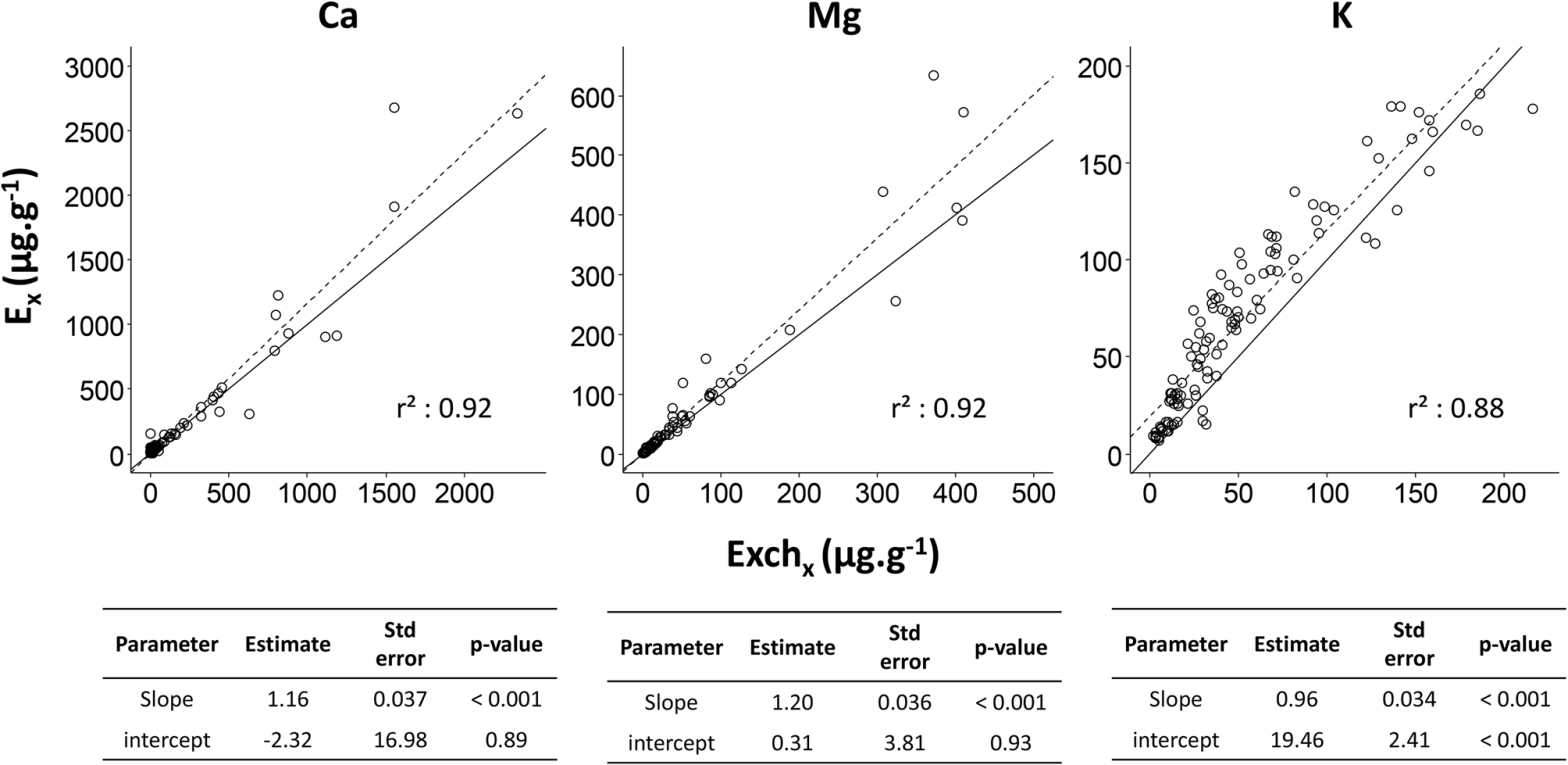
Relationship between isotopically exchangeable (E_x_) and conventional exchangeable (Exch_x_) pools for Ca, Mg and K. Linear regression parameters are given in each figure. Solid and dotted lines represent, respectively, y:x = 1 and linear regression.

Strong linear relationships were found between isotopically exchangeable pools and conventional exchangeable pools (Fig. 3). Correlation coefficients were respectively 0.92, 0.92 and 0.88 for Ca, Mg and K (p-values < 0.001). Linear regressions slopes were greater than the 1:1 slope for Ca and Mg whereas for K, linear regression slope was similar to the 1:1 slope but intercept was different. No other significant relationships were found between E_x_ pools of Ca, Mg and K and experimental data or soil physico-chemical properties over the entire dataset.

### Tracer recovery in the different soil pool

**Figure 4:**
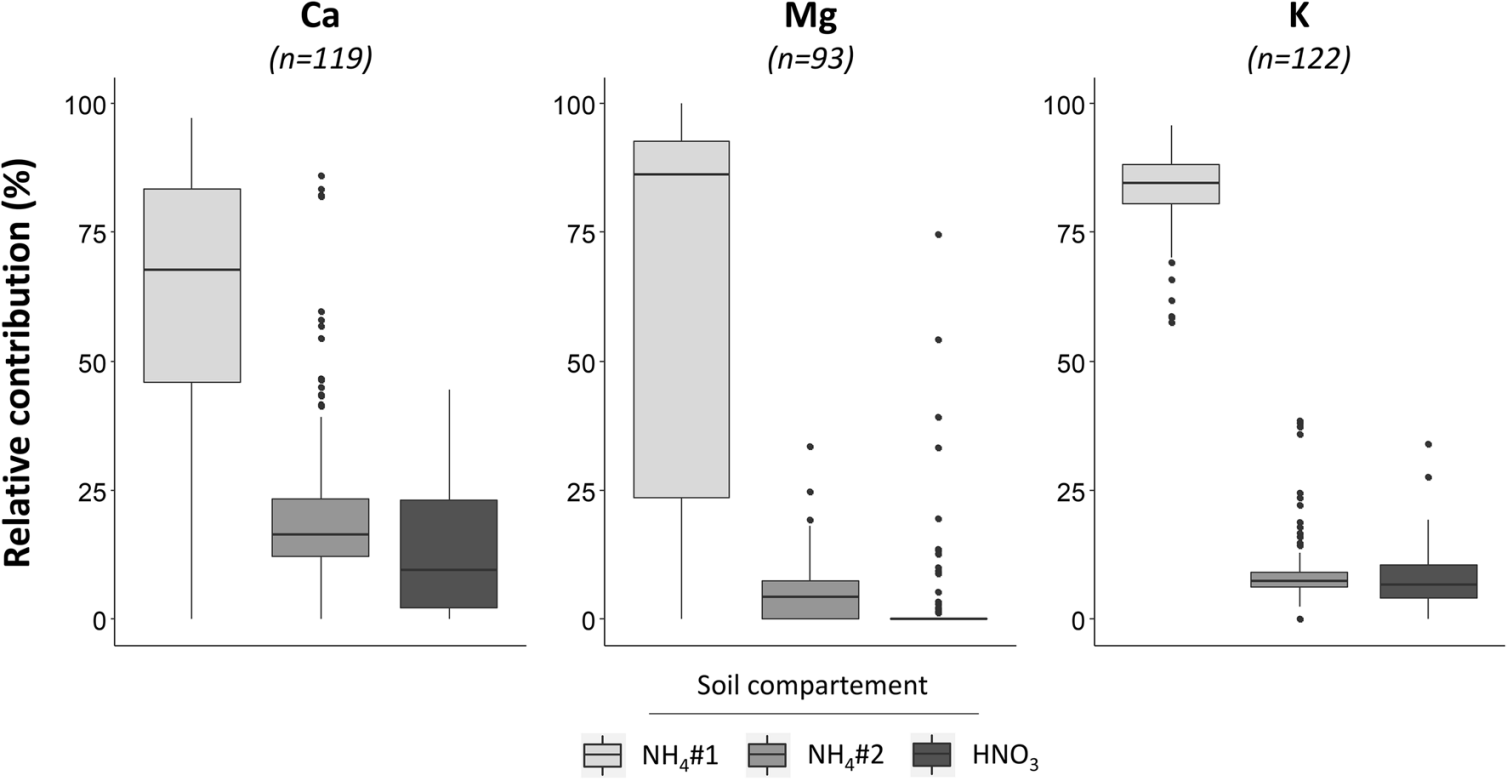
Relative contribution (%) of each extracted soil pool (NH_4_#1, NH_4_#2 and HNO_3_) to the isotopically exchangeable pool (Ex) for Ca, Mg and K. The number of validated samples (n) is given in brackets.

For all three elements, most of the tracer amounts recovered in the soil pools were found in the NH_4_ extracted pools (Fig. 4, for individual results Table S4 supplementary): the median relative contribution for NH_4_#1 and NH_4_#2 was 68% and 17% for Ca, 90% and 5% for Mg and 85% and 7% for K, respectively. For Ca and Mg, the median relative contribution in the three pools was as follows: NH_4_#1 > NH_4_#2 > HNO_3_. Potassium was similar to Ca and Mg but the median relative contribution in the NH_4_#2 pool was close to that in the HNO_3_ pool (~ 7%). The relative contribution in the NH_4_#1 pool showed higher variability for Ca and Mg compared to the other soil pools, the interquartile was 37% and 71%, respectively, whereas only 7% for K.

Analytically significant amounts of isotope tracers were found in the HNO_3_-extracted pool for almost all samples for Ca and K: 95% and 96% respectively whereas only 19% for Mg (Fig. 4). For all three elements, the relative contribution of the HNO_3_ extracted pool was lower than in the NH_4_ extracted pools: the median relative contribution was 10%, 0% and 7% for Ca, Mg and K, respectively, and the maximum value across soil samples reached 45% for Ca, 74% for Mg and 34% for K.

**Figure 5:**
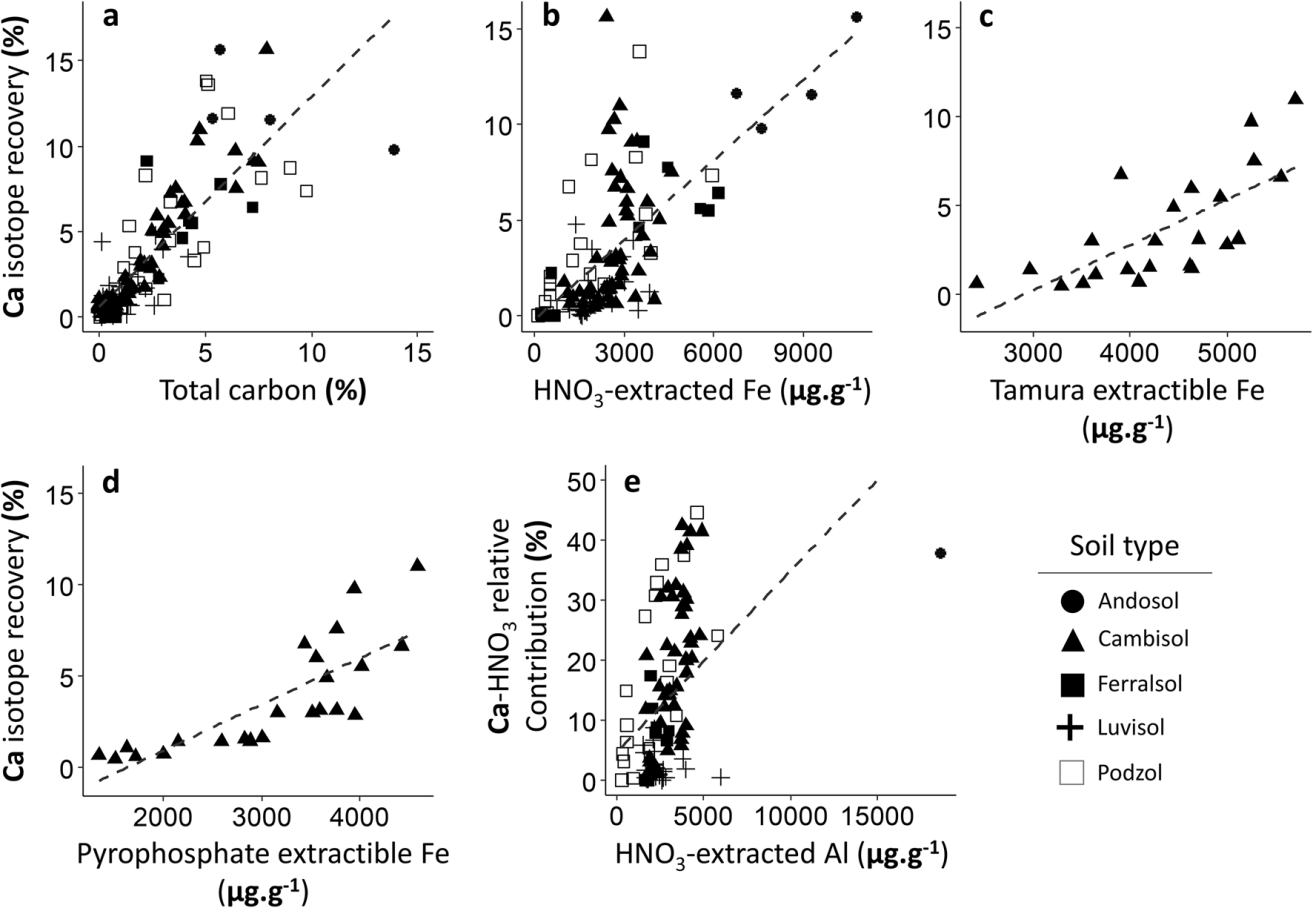
Relationship between the isotopic recovery of Ca in the HNO3-extrated pool and (**a**) total carbon, (**b**) Fe HNO3-extracted pool, (**c**) Tamura and (**d**) pyrophosphate extractible Fe. (**e**) shows the relationship between the relative contribution of the Ca HNO3-extracted pool and the Al HNO3-extracted pool. The linear regressions are represented by the dotted lines.

Significant relationships were found between the Ca-tracer recovery in the HNO_3_-extracted and the total carbon content in the soil sample, as well as with HNO_3_-extracted iron content (Fig. 5a,b). Correlation coefficients were 0.65 and 0.42 respectively (p-value < 0.001). For Breuil samples, significant relationships were also found between the Ca-tracer recovery in the HNO_3_-extracted pool with Tamura and Pyrophosphate extractible Fe (Fig. 5c,d). Correlation coefficients were 0.48 and 0.62 respectively (p-value < 0.001). To a lesser extent, the relative contribution of Ca HNO_3_-extracted pool to E_Ca_ was significantly correlated to the HNO3-extracted pool of Al (correlation coefficient of 0.17 and p-value < 0.001) (Fig. 5e).

**Figure 6:**
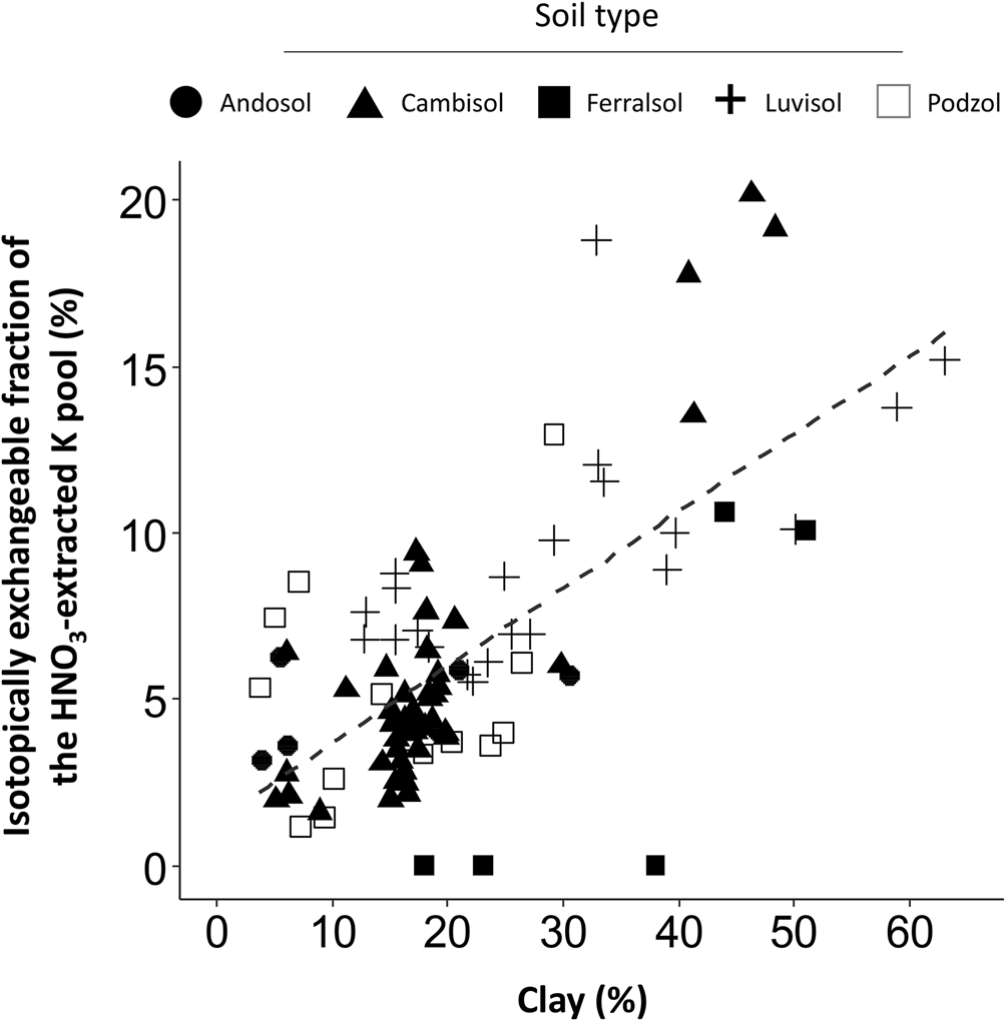
Relationship between the pourcentage of clay (texture class < 2 µm) in soil samples and the isotopically exchangeable fraction of the HNO3-extracted K pool. The linear regression is represented by the dotted line.

A significant relationship was found between the isotopically exchangeable fraction of the HNO_3_-extracted pool of K and the clay content in soil samples (r-squared = 0.47, p-value < 0.001) (Fig. 6), but the relationship was not significant for Ca and Mg.

## Discussion

### Isotopically exchangeable pools

Our results show over the whole dataset, for the majority of these samples, that the isotopically exchangeable pools (E_X_) of Ca, Mg and K are larger than the conventional exchangeable pool (Exch_X_). The majority of samples showed an E_X_:Exch_X_ ratio greater than 1 for Ca, Mg and K. For a substantial proportion of the dataset (43%, 27% and 47% respectively for Ca, Mg and K), E_x_ pools were more than 50% larger than the Exch_X_ pools. This trend was confirmed by a Wilcoxon–Mann–Whitney comparison test that showed that isotopically exchangeable pools were significantly larger than their respective exchangeable pool, though homoscedasticity between the compared pools was not met for Mg. Our results are consistent with previous studies for Ca and K. Blum and Smith^41^ showed, in an isotopic dilution assay using the ^45^Ca radiogenic isotope over 16 different type of soils, that E_Ca_ was significantly greater than Exch_Ca_ for 6 soil samples, lower for 2 samples and not significantly different for 8 samples. Reeve et al.^43^ showed that 5 soil samples out of 7 showed an E_Ca_ pool greater than the Exch_Ca_ pool. For K, apart from the study by Graham and Fox^46^ in which 4 out of the 11 studied soil samples showed an E_K_ pool lower than the Exch_K_ pool, all other studies showed E_K_ pools systematically greater than the Exch_K_ pools^38–40^.

For a limited proportion of the dataset, the E_x_ pool was smaller than the Exch_X_ pool. This result was unexpected because the conventional exchangeable pool is assumed to be in rapid equilibrium with soil solution^2,3^. The isotopically exchangeable pool was thus expected to be at least equal to the exchangeable pool. However, for the majority of the samples of this study, as reported in previous assays^44,45^, only a fraction of the exchangeable pool contributed to the isotopic equilibrium (the isotopically exchangeable proportion of the NH_4_#1 and NH_4_#2 extracted pools were (median): 68% and 54% for Ca, 81% and 28% for Mg, and 88% and 53% for K). That less than 100% of the exchangeable pool reached isotopic equilibrium with the isotopically labelled solution is most likely due to the fact that certain sites that are extractible with a highly concentrated reagent may not be exchangeable with a dilute solution^47^. The isotopically exchangeable proportion of the NH_4_#1 and NH_4_#2 extracted pools of Ca, Mg and K was non linearly and positively correlated to their respective conventional exchangeable pool. This result agrees with the mechanisms of ion exchange by which the likelihood of a given dissolved cation to undergo exchange with a cation adsorbed on the cationic exchange complex is proportional to the abundance of the adsorbed cation on the cationic exchange complex^48^.

Despite these results, for all samples showing E_x_ lower or similar in size to Exch_x_, analytically significant amounts of isotope tracer were recovered in the HNO_3_-extracted soil pool (Table S3 supplementary). Hence, for these samples as well, the results of this study show that soil phases other than the conventional exchangeable pool directly contribute to the geochemical equilibrium processes for Ca, Mg and K. These geochemically reactive pools of Ca, Mg and K unaccounted for by conventional soil analysis methods are likely to play a very important role in the biogeochemically functioning of forest ecosystems, most particularly in soils where the conventional exchangeable pools are low (Fig. 2).

### Storage forms

Unsurprisingly, the isotopically exchangeable pools of Ca, Mg and K were mainly composed of NH_4_-exchangeable pools of Ca, Mg and K and their variability was mainly explained by the Ca, Mg and K exchangeable pool variability (Fig. 3). Nevertheless, for the great majority of the dataset, isotope tracers were also recovered in the HNO_3_-extracted pool. The amounts of isotope tracer recovered in this pool were analytically significant and, for numerous samples, substantial (Fig. 4). It is unlikely that this recovery may be explained by residuals amounts of isotope tracer after the two AcONH_4_ extractions (NH_4_#1 and NH_4_#2) because an intermediary rinsing extraction was performed. Concentrations of nutrient cations measured in this rinsing extraction were generally low and often too low (for 41%, 51% and 91% of samples for Ca, Mg and K, respectively) for isotopic analysis.

#### Calcium

The HNO_3_-extracted pool of Ca accounted for a significant proportion of the measured E_Ca_ pool (Fig. 4), and was larger for Ca compared to Mg and K. The Ca-tracer recovery in the HNO_3_-extracted pool was strongly correlated to the soil carbon content (R^2^ = 0.65, p-value < 0.001) (Fig. 5a) thus suggesting that significant amounts of Ca adsorbed or chelated to organic compounds in the soil contribute directly to geochemical equilibrium reactions but are not extracted by conventional exchangeable cation extractions. This may be explained by the much higher affinity of organic compounds for Ca compared to most other cations such as NH_4_^+^^49–51^.

A strong and significant correlation was also found between the Ca-tracer recovery in the HNO_3_-extracted pool and the HNO_3_-extracted pool of Fe (R^2^ = 0.42, p-value < 0.001) (Fig. 5b). For the Breuil samples, Tamura-extracted and pyrophosphate-extracted Fe (Fig. 5c,d) were also correlated to Ca-tracer recovery in the HNO_3_-extracted pool (r^2^ 0.48 and 0.62 respectively for Tamura and pyrophosphate. p-values < 0.01). The Ca HNO_3_-extracted pool relative contribution was also significantly correlated to the HNO_3_-extracted pool of Al (R^2^ = 0.17, p-value < 0.001) (Fig. 5e). Because these different soil extractions mainly dissolve Fe and Al (hydr)oxides, it is likely that these amorphous secondary minerals act as a support to a pool of isotopically exchangeable Ca. In agreement with these results, the greatest E_Ca_:Exch_Ca_ ratios were found in podzol and alumic cambisol soil samples where the soil organic matter and Fe and Al (hydr)oxides are abundant and likely to play an important role in calcium biogeochemistry.

Iron and Aluminium (hydr)oxides are common in soils^52^ and occur as amorphous minerals ranging from short-range-ordered to increasingly crystalline phases. The point of zero charge (pzc) of synthetic Fe and Al (hydr)oxides is generally above 6^53^. Yet in natural environment, Fe and Al (hydro)oxides pzc are lower^54^ so that negative charges could be developed at pH_water_ < 6 and direct adsorption of Ca on such minerals has been demonstrated for the range of soil pH in our soil samples (pH_H2O_ range 3.4–6.2, median 4.6)^29,55–57^. However direct adsorption is unlikely to be the main storage mechanism for the isotopically exchangeable Ca in the HNO_3_-extracted pool. Instead, the results of our study strongly suggest that geochemically reactive Ca is retained on the surface of Al and Fe (hydr)oxide minerals through an anion-bridge such as sulfate or organic acids as suggested by previous studies^32,45,58^.

Van der Heijden et al.^44,45^ in a similar isotopic dilution experiment carried out on an alumic cambisol soil, showed a non-negligible contribution of Ca, Mg and K pools extracted with a Tamm reagent and with a HNO_3_ reagent (1 mol L^−1^) to the isotopically exchangeable pools. They suggested that the main storage form of Ca and Mg in the Tamm and HNO_3_-extracted pools was cations indirectly adsorbed on Al and Fe oxides and hydroxides through (i) P or organic acid-mediated bridging or (ii) occluded within Fe and Al phases or their organic co-precipitates. Additionally, Hall and Huang^32^ showed the role of occluded cations in sustaining plant nutrition through Fe-(hydr)oxide reduction, whereby these occluded cations may act as a bank to replenish exchangeable pools on timescales of hours to months. Iron and aluminium (hydr)oxides are thus likely to be the support of a geochemically reactive HNO_3_ pool of Ca which is not extracted by conventional exchangeable cation methods. This may be explained by possible temporary occlusion of Ca in these soil phases: occluded in supramolecular aggregates where various large organic molecules are held together by van der Waals forces, hydrogen bonds and metal bridging involving Ca and Fe-hydr(oxides)^59–61^. These amorphous minerals are known to very dynamically precipitate and dissolve over time as a result of changes in the physical and chemical properties of the soil solution^31^ and under the influence of microbial and plant activity^32^.

#### Potassium

Similar to Ca, K tracer was recovered in the HNO_3_-extracted pool for 97% of all validated samples and represented on average 8% of the isotopically exchangeable pool. A strong linear and positive correlation was found between the clay content and the isotopically exchangeable fraction of the HNO_3_ extracted K pool (R^2^ = 0.52; p < 0.001) (Fig. 6). This relation is most likely explained by the presence of “fixed” potassium (K-specific exchange sites) in phyllosilicates^33^, held between adjacent tetrahedral phyllosilicate layers of micas and 2:1 clay minerals such as vermiculite or illite, but not extractible with concentrated salt extractions such as NH_4_^+^^62,63^. K specific ion-exchange reactions between the soil solution and the clay-interlayer potassium pools may have occurred during the isotopic equilibrium stage of the experiment. The HNO_3_ reagent (1 mol L^−1^) is likely to have caused a sufficient weathering of the phyllosilicates and a subsequent release of interlayer potassium. However, quantitative phyllosilicate mineralogy would be necessary to better characterize the role of clay minerals and K-specific exchange sites because (1) not all phyllosilicates contain pools of interlayer potassium and (2) the pools of interlayer K may respond very differently depending on the phyllosilicate. For instance, at the Breuil site where quantitative mineralogy was available^64^, a positive correlation was found between the vermiculite content with K tracer recovery, the relative contribution of the HNO_3_-extracted K pool to E_K_ and the E_K_:Exch_K_ ratio. By contrast, no correlations were found with the kaolinite (1:1 clay mineral) or illite (2:1 clay mineral) contents (data not shown).

The highest relative contribution of the HNO_3_-extracted pool of K and E_K_:Exch_K_ ratios were however found for the andosol and podzol soil types where the clay content is low. It is thus likely that other forms of storage in addition to interlayer K contribute to supporting the isotopically exchangeable pools of K. Relationships in our dataset suggest that potassium may also be adsorbed through ion-exchange processes on the surface of amorphous silica gels and aluminosilicates. For the Breuil-Chenue site, a positive correlation was found between, on the one hand, the difference between E_K_ and Exch_K_ and, on the other hand, the difference between the Tamm-extracted K pool and Exch_K_ (R^2^ = 0.36, p < 0.001) (data not shown). The Tamm reagent, although non-selective, primarily dissolves (acid dissolution and chelation) poorly crystallized Al and Fe (hydr)oxides and amorphous aluminosilicates. These adsorption sites may be K-specific or occluded and thus not accounted for by conventional exchangeable cation pool extractions. A previous isotopic dilution assay also suggested that Tamm labile pools of K were mainly linked to amorphous aluminosilicate phases^45^.

#### Magnesium

In contrast with Ca and K, analytically significant amounts of Mg tracer were only found in the HNO_3_-extracted pool in 19% of the dataset. However, it is likely that this difference with Ca and K is not or not solely the result of a very contrasting behaviour of Mg in the geochemical equilibrium processes. Indeed, a previous study using a similar isotopic dilution approach showed that soil phases extracted with Tamm and HNO_3_ reagents significantly contributed (up to 11%) to the Mg geochemical equilibrium between the solution and the soil^44^ in the Breuil-Chenue soil profile. It is most likely that the behaviour of Mg observed in the current isotopic dilution assay is due to the experimental design: the quantity of isotopically enriched Mg was probably too small to efficiently isotopically label soil phases other than the exchangeable pool. Compared to Ca and K, in the majority of samples, large amounts of Mg were extracted from the soil during the HNO_3_-extraction step most likely due to the dissolution of Mg-bearing soil minerals (biotite, muscovite, vermiculite, chlorite, etc.) making isotope tracer recovery difficult to analytically resolve (high isotopic dilution). Indeed, the ratio of the amount of isotope tracer applied over the HNO_3_-extracted pool size was much lower for Mg (0.074), than for Ca (1.33) or K (0.246). This demonstrates the importance of the experimental parameters when setting up and the limits of isotopic dilution assays: the concentration of the tracing solution should be as close as possible as in situ concentrations and in the same time, the amount of applied tracer should be sufficiently high to ensure the isotopic labelling of the different studied soil phases.

For the limited number of samples for which Mg isotope tracer was recovered in the HNO_3_ extracted Mg pool, no significant relations were found with the other variables of the dataset. However, the highest E_Mg_:Exch_Mg_ ratios were found in andosol and podzol soil types similarly to both Ca and K. In a previous isotopic dilution assay^45^, geochemically reactive Mg was shown to be stored in Tamm and HNO3 extracted soil phases in forms close to those previously discussed for Ca: adsorbed to Al and Fe (hydr)oxide secondary minerals through anion-bridges.

### Implication at the soil profile scale

**Figure 7:**
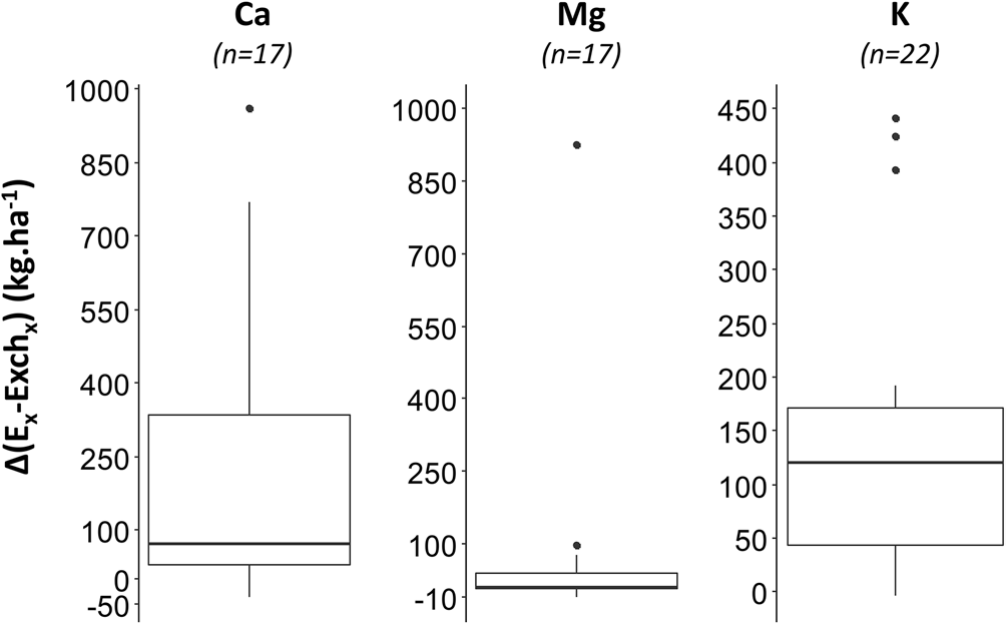
Boxplot distribution of the differences between Ex and Exchx stocks at the soil profile scale for Ca, Mg and K. The number of soil profiles (n) is given in brackets.

Nearly all sites had E_x_ soil profile stocks (kg ha^−1^) greater than the Exch_x_ stocks (respectively 82%, 94% and 95% of samples for Ca, Mg and K) (Table S5 supplementary). For calcium, E_Ca_ stocks were two-fold greater than Exch_Ca_ stocks for nearly half of the sites (47%), three-fold greater for 24% of the sites. Comparatively, less sites had E_Mg_ and E_k_ stocks which were at least two-fold greater than Exch_Mg_ and Exch_K_ (6% and 32%, respectively). The relative differences between E_X_ and Exch_X_ stocks were greatest for the sites with low exchangeable pools of Ca, Mg and K. Differences represented in median + 73 kg ha^−1^ for Ca (range from − 37 to + 960 kg ha^−1^), + 12 kg ha^−1^ (from − 9 to + 927 kg ha^−1^) for Mg and + 121 kg ha^−1^ for K (from − 3 to + 441 kg ha^−1^) (Fig. 7). These results highlight that at the soil profile scale, the conventional exchangeable pools may greatly underestimate the pool of cations that contribute to geochemical equilibrium between the soil and the solution and thus to the plant-available pools.

In the European beech (*Fagus sylvatica* L.) plot of the Breuil-Chenue experimental site, nutrient input–output budgets predicted a depletion of the exchangeable pools of Ca (3.1 kg ha^−1^ year^−1^) and Mg (0.8 kg ha^−1^ year^−1^) in the soil profile. Evidence from an isotopic tracing experiment^28^ concurred with the predicted Mg depletion but showed that the exchangeable pools of Ca had not decreased. The present study shows that isotopically exchangeable pool of Ca (0–70 cm) was much greater (138 kg ha^−1^) than the exchangeable pool (73 kg ha^−1^) whereas the isotopically exchangeable and exchangeable pools of Mg were similar (35 and 33 kg ha^−1^, respectively). It is most likely that the unaccounted pool of isotopically exchangeable Ca has contributed to buffering the depletion of the conventional exchangeable pools over time.

Plant driven processes in specific soil zones can also increase the stocks of plant-available nutrient cations. The consequences of biogeochemical processes on nutrient release within the rhizosphere of plant roots are well documented^65^. A recent study at the Itatinga experimental site (Brazil) sampled in our study suggested that root-induced weathering of K-bearing minerals, partly related to enhanced rhizosphere acidification could explain the observed increase in exchangeable K concentration within the rhizosphere of *Eucalyptus grandis* trees^66^.

## Conclusion

**Figure 8:**
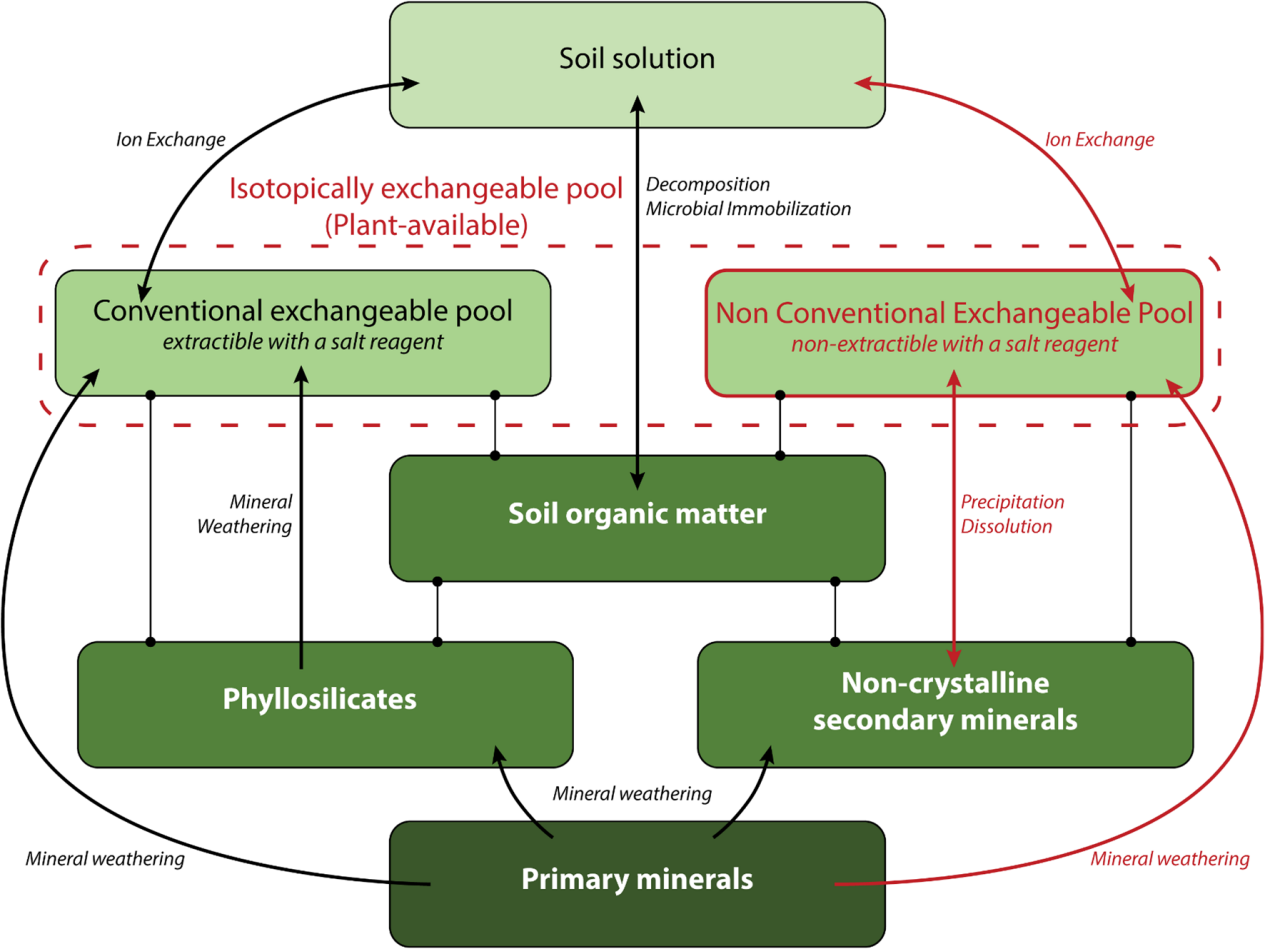
Conceptual model of Ca, Mg and K plant-availability. Dot-ended lines symbolize a support function. Black boxes and arrows symbolize the pools and fluxes of nutrient cations that are taken in the current conceptual model. Red boxes and arrows represent the pools and fluxes evidenced by the present study.

This study validated, for a wide variety of forest soils, the hypothesis that pools of Ca, Mg and K in the soil in addition to the exchangeable pools contribute directly and on short time scales to the geochemical equilibrium processes between the soil and solution. Although the isotopically exchangeable pools of Ca, Mg and K vary widely between the different soil samples, these pools are in many cases substantially greater compared to their respective conventional exchangeable pool. The differences between the E_X_ and Exch_X_ pools were most remarkable for Ca and K, and lesser for Mg. These previously unaccounted pools of Mg, Ca and K in the soil fertility diagnostic are most likely to play an essential role in the biogeochemical functioning of forest ecosystems, in particular in ecosystems where the resources of Ca, Mg and K are low, by providing a supplementary buffer capacity to the depletion of cations. These groundbreaking results enable to reframe the conceptual model of plant available pool by integrating this additional pool of available nutrient cations (Fig. 8).

Soil phases extracted with weak to strong acid dissolution extraction methods are likely to support source and sink pools of Ca, K and possibly Mg. Hypotheses of the nature of these soil phases may be formulated. The isotopically exchangeable pool of Ca appears to be associated with amorphous and poorly crystalized secondary minerals through interactions with soil organic matter, whereas the isotopically exchangeable pool of K is likely associated with K-specific sites of phyllosilicates and amorphous aluminosilicates. These soil phases in addition to being a support for plant-available cations over short time scales may also significantly contribute to the long-term plant-availability as a source of cations released by their mineral weathering.

This study shows that the use of stable isotopic tracers to quantify the plant-available pools of Ca, Mg, and K on short time scales (source and sink pools) is both adequate and relevant in order to better understand biogeochemical cycling and tree nutrition in forest ecosystems. Although the precise identification and characterization of the soil phases that support the geochemically reactive pools and their interactions is a challenge, it is a vital step to better understanding and quantifying their role in soil geochemistry and stable isotope approaches are a powerful tool to achieve this goal.

## Material and methods

### Study sites

Twenty-six sites from 4 countries were selected amongst long-term forest monitoring networks (French ICP-forests level II sites and Swedish ICP-Integrated Monitoring sites), ANAEE/IN-SYLVA experimental sites as well as the Luquillo Critical Zone Observatory in Puerto Rico (Table S1 supplementary) in order to cover a range of acidic (pH_water_ < 6) and non-hydromorphic soils from a wide variety of climatic, edaphic, chemical fertility and tree species cover conditions. The scope of this study focuses on acidic soils as they are representative of a large proportion of forest ecosystems and are particularly sensitive to disturbances. When several replicates of soil profiles were sampled in each plot, a composite soil profile was established from the archived soil samples. Depending on the number of sampled soil layers, a maximum of 5 soil layers covering the entire available profile were selected for each plot. Each plot was covered by one dominant tree species. All sites, except the Swedish IM sites which are located in natural reserves, followed conventional and local forest management. The dataset contained no fertilized plots apart from Itatinga (Brazil), where background fertilization was added in all plots for every rotation similar to commercial plantations (300 kg ha^−1^ NPK—10:20:10). The global dataset was composed of 143 individual samples and encompasses 5 different types of soil, 11 tree species and 3 climatic zones.

The dataset was compiled from different databases encompassing soil physical and chemical properties measured in different laboratories following different protocols (Table S2 supplementary). The soil physical and chemical property dataset included particle size content distribution (i.e. clay, silt, sand), bulk density, total carbon and total nitrogen measured by wet combustion (Kjeldhal method for N; Walkey and Black or Anne method for C) or dry combustion and exchangeable cations (NH_4_Cl, KCl, BaCl_2_, NH_4_OAc or cobaltihexamine), noted Exch_Ca_, Exch_Mg_ and Exch_K_. The sum of exchangeable base cations (S) and exchange acidity (EA) were respectively defined as the sum of exchangeable Ca, Mg and K and as the sum of exchangeable Al and protons. In addition, specific extractions of soil phases were included, in particular Tamm, Tamura, Mehra-Jackson and pyrophosphate extractions.

### Isotopically exchangeable pools of Mg, Ca and K

The stable isotopic dilution technique was used to quantify the pools of Ca, Mg and K stored in the soil that may exchange rapidly with ions of the same element in the soil solution, so as to replace these ions in solution as they become lost from the system through plant uptake, leaching, or other output fluxes (isotopically exchangeable pool noted E_Ca_, E_Mg_ and E_K_).

A ^44^Ca, ^26^Mg and ^41^K labelled solution (concentration: 800, 200 and 500 μg L^−1^ for Ca, Mg and K respectively) was made up from dissolved ^44^CaCO_3_ (96.45 atom% 44Ca), ^26^MgO (99.25 at% ^26^Mg) and ^41^KCl (97 at% ^41^K). The pH_water_ of the labelled solution was adjusted with purified nitric acid to the soil pH_H2O_ of each sample. For each soil sample, 2.50 g of 2 mm-sieved dry soil were placed in a 50 mL polypropylene tube and 50 mL of the labelled solution were introduced. Tubes were immediately caped and placed in a continuous shaker. After 1 h, 6 h, 24 h and 48 h, the tubes were centrifuged (3000 rot min^−1^) during 20 min to sample a 12.5 mL aliquot of the supernatant solution for chemical and isotopic analyses. Tubes were then vortexed and replaced in the continuous shaker until the following time step. The isotopic variation in the solution enables to quantify the isotopically exchangeable pools of Ca, Mg and K at different stages of the equilibrium process.

After the 48 h time step, a four-stage sequential soil extraction protocol was conducted:

1. 50 mL of 1 mol L^−1^ ammonium acetate (unbuffered) continuously shaken during 1 h. Hereafter referred as NH_4_#1.
2. 50 mL of 1 mol L^−1^ ammonium acetate during (unbuffered) continuously shaken during 24 h. Hereafter referred as NH_4_#2.
3. 50 mL of 0.1 mol L^−1^ ammonium acetate at pH 3 (nitric acid) continuously shaken during 3 h.
4. 50 mL of 1 mol L^−1^ nitric acid continuously shaken during 20 h. Hereafter referred as HNO_3_.

Ammonium acetate extracts the pools of cations stored in an ion-exchangeable form. The 0.1 mol L^−1^ ammonium acetate stage (at pH 3) was included as a rinsing step between the ammonium acetate and HNO_3_ extractions and ensured that all exchangeable cations were extracted before moving on to the 1 mol L^−1^ nitric acid stage which is a strong non-selective extraction capable of dissolving many secondary mineral phases such allophane, iron and aluminium organometallic complexes, some part of hydrated iron and aluminium oxides, clay minerals and readily weathered primary minerals^67^.

#### Sample analysis

Major element concentrations were measured by ICP-AES (AGILENT 7500 series). ^44/40^Ca, ^26/24^Mg and ^41/39^K isotope ratios were measured by ICP-MS (Analytik Jena 820MS). Instrument optimization and methods are detailed in van der Heijden et al.^45^. All samples were diluted or evaporated to the same concentration: 100 µg L^−1^ Mg, 100 µg L^−1^ Ca, and 200 µg L^−1^ K. Ammonium acetate was digested prior to isotopic analysis with 5 mL hydrogen peroxide (H_2_O_2_ 30%) after complete sample evaporation. Instrumental mass discrimination was corrected using the standard bracketing technique by measuring standards of known isotopic composition every 12 samples: the bracketing standards were selected to ensure that enrichment of each sample fell between two standards. The tracer detection limit was set at 10‰ for ^26^Mg and ^44^Ca and 20‰ for ^41^K^45^. Tracer recovery was also considered below the detection limit when the sample elemental concentrations were too low to conduct isotope ratio analysis.

### Calculation methodology

#### Calculation methodology

The isotopically exchangeable pool is in isotopic equilibrium with the soil solution. The quantification of this pool is thus based on the assumption that its isotopic composition is equal to that measured in the solution using (shown for Mg):

1 $${E}_{Mg}= {Mg}_{label}\times \left(\frac{1}{{\alpha }_{solution}^{Mg}}-1\right)$$

where ${E}_{Mg}$ is the isotopically exchangeable pool (µg g dry soil^−1^), ${Mg}_{label}$ is the amount of isotopically enriched Mg added into the system (µg g dry soil^−1^) and ${\alpha }_{solution}^{Mg}$ is the fraction of Mg in the solution originating from the initial tracing solution, calculated as:

2 $$ \alpha_{solution}^{Mg} = \frac{{(\%^{26} Mg)_{solution} - (\%^{26} Mg)_{nat} }}{{(\%^{26} Mg)_{label} - (\%^{26} Mg)_{nat} }} $$

where $(\%^{26} Mg)_{solution}$ is the ^26^Mg atomic abundance in the solution, $(\%^{26} Mg)_{nat}$ is the natural ^26^Mg atomic abundance, and $(\%^{26} Mg)_{label}$ is the ^26^Mg atomic abundance of the initial tracing solution.

The relative distribution of the stable isotope tracers in the different extracted pools of the soil was calculated as the amount of Ca, Mg and K originating from the tracing solution (tracer recovery) in each pool divided by the sum of tracer recovered in all three extractions, and quantifies the relative contribution of each extracted soil pool (i.e. NH_4_#1, NH_4_#2 and HNO_3_) to the isotopically exchangeable pool of Ca, Mg and K.

The isotopically exchangeable fraction of each extracted soil pool (the proportion of each pool that is isotopically exchangeable) was calculated by assuming that its isotopic composition is equal to that of the solution:

3 $$ \alpha_{pool}^{IE Mg} = \frac{{(\%^{26} Mg)_{pool} - (\%^{26} Mg)_{nat} }}{{(\%^{26} Mg)_{solution} - (\%^{26} Mg)_{nat} }} $$

where $(\%^{26} Mg)_{pool}$ is the ^26^Mg atomic abundance in the extracted pool and $(\%^{26} Mg)_{solution}$ the fraction of Mg in the solution originating from the initial enriched tracing solution at the last time step of the isotopic dilution experiment (i.e. 48 h).

#### Sample validation

Because isotopically enriched samples are sensitive to contamination; the isotopic dilution results for each soil sample were verified and samples were excluded from the dataset according to the following criteria.

- E_Ca_, E_Mg_ and E_K_ data. Samples displaying missing or contaminated data for one or more time steps during the isotopic dilution experiment were excluded.
- Soil extraction data. Samples were excluded if one or more of the 3 extractions (i.e. NH_4_#1, NH_4_#2 and HNO_3_) was missing or if the cumulated tracer recovery in the whole system (soil + solution) was above 120%.

After data validation, the E_Ca_, E_Mg_ and E_K_ dataset was composed of 83 samples for Ca (22 sites), 90 samples for Mg (24 sites), and 114 samples for K (24 sites) over a global dataset of 143 samples. The soil extraction dataset was composed of 119 samples for Ca (26 sites), 93 samples for Mg (23 sites), and 122 samples for K (25 sites).

#### Statistical methods

Statistical analyses were computed using R version 3.6.1^68^ (https://www.r-project.org/).

The difference between E_x_ and Exch_x_ were tested over the entire dataset with a non-parametric Wilcoxon–Mann–Whitney signed rank test for Ca, Mg and K. E_x_ and Exch_x_ normal distribution was tested with a Shapiro–Wilk test and homoscedasticity was tested with a Levene test. Homoscedasticity was met for Ca and K but not for Mg (p-value < 0.05).

The repeatability of E_x_ measurements was estimated by double replicates for 55 samples and the E_X_:Exch_X_ ratio was calculated for each replicate. The mean relative error for this replicated dataset was 16%, 14% and 8% for Ca, Mg and K, respectively. A sample was defined as significantly greater than Exch_x_ if E_x_:Exch_x_ ratio was greater than the respective mean relative error (i.e. if E_x_:Exch_x_ greater than 1.16 for Ca, 1.14 for Mg and 1.08 for K).

Correlations between the different variables of the dataset were tested with a Spearman correlation test. Only statistically significant correlations are presented.

## Supplementary information

Supplementary Table S1.

Supplementary Table S2.

Supplementary Table S3.

Supplementary Table S4.

Supplementary Table S5.
