# Author Correction: Conventional analysis methods underestimate the plant-available pools of calcium, magnesium and potassium in forest soils

**DOI:** 10.1038/s41598-021-81982-2

**Published:** 2021-02-16

**Authors:** Jérémie Bel, Arnaud Legout, Laurent Saint-André, Steven J. Hall, Stefan Löfgren, Jean-Paul Laclau, Gregory van der Heijden

**Affiliations:** 1 INRAE Grand-EST Nancy, UR 1138 Biogéochimie Des Ecosystèmes Forestiers, Route d’Amance, 54280 Champenoux, France; 2 Department of Ecology, Evolution, and Organismal Biology, Iowa State University, 251 Bessey Hall, Ames, IA 50011 USA; 3 Department of Aquatic Sciences and Assessment, Swedish University of Agricultural Sciences, P.O. Box 7050, 750 07 Uppsala, Sweden; 4 CIRAD, UMR 210 ecologie fonctionnelle et biogéochimie des sols et des agro-écosystèmes, Campus SupAgro, Bâtiment 12–2 place Viala, 34060 Montpellier, Cedex 2, France

Correction to: *Scientific Reports* 10.1038/s41598-020-72741-w, published online 24 September 2020

The original version of this Article contained errors in the spelling of the authors Jérémie Bel, Arnaud Legout, Laurent Saint-André, Steven J. Hall, Stefan Löfgren, Jean-Paul Laclau & Gregory van der Heijden which were incorrectly given as Bel Jérémie, Legout Arnaud, Saint-André Laurent, J. Hall Steven, Löfgren Stefan, Laclau Jean-Paul & van der Heijden Gregory.

These errors have now been corrected in the PDF and HTML versions of the Article.

